# Study on the Clinical Safe and Effective Methods of Arsenic-Containing Compound-Qinghuang Powder in the Treatment of Myelodysplastic Syndrome

**DOI:** 10.1155/2017/2095682

**Published:** 2017-11-16

**Authors:** Qianzhe Zhu, Zhongyang Deng, Shirong Zhu, Pan Zhao, Mingjing Wang, Xiaomei Hu

**Affiliations:** Department of Hematology, Xiyuan Hospital, China Academy of Chinese Medical Sciences, Beijing 100091, China

## Abstract

**Objective:**

To establish the clinical safe and effective methods of arsenic-containing compound-Qinghuang Powder (compound-QHP) in the treatment of myelodysplastic syndrome (MDS).

**Methods:**

200 patients with MDS were treated with compound-QHP (daily dose of 0.1 g realgar). The blood arsenic concentrations (BACs) were detected by atomic fluorescence spectrophotometry (HF-AFS). After treatment for 1 month, the patients were randomly divided into group A and group B when the BACs were less than 20 *μ*g/L. Daily dose of realgar was maintained in group A and it was increased to that when the BACs were more than 20 *μ*g/L in group B. The BAC and clinical efficacy and safety in two groups were compared at the end of the treatment with compound-QHP.

**Results:**

The average BAC of group B was significantly higher than that of group A (*P* < 0.01). The rates of hematology improvement and reduced transfusion were significantly higher in group B than in group A (*P* < 0.05). The HGB, ANC, and PLT significantly increased in group B after treatment (*P* > 0.05).

**Conclusions:**

Monitoring the BAC and adjusting the daily dose of realgar to increase the effective BAC and then improving efficacy without increasing the clinical toxicity are the clinical safe and effective methods in the treatment of MDS.

## 1. Introduction

Myelodysplastic syndrome (MDS) is a clonal disorder of* hematopoietic* stem or progenitor cell which is characterized by ineffective hematopoiesis of the bone marrow, long-term progressively refractory anemia, and frequent development of leukemia [1].

Qinghuang Powder (QHP) is made of* indigo naturalis* and* realgar* and realgar is an arsenic-containing mineral with toxicity. Our previous study had reported that QHP was effective in treating MDS [2, 3], and the clinical efficacy was related to the blood arsenic concentrations when they are higher than 20 *μ*g/L [4]. Low arsenic concentration of blood of patients with QHP has poor treatment effect. Compound-Qinghuang Powder (compound-QHP) was a modified prescription based on the traditional QHP that could better ensure the blood arsenic concentrations by decreasing the side digestive effect [5, 6]. Our study tested the blood arsenic concentrations of patients with MDS and increased the dosage of realgar for patients whose blood arsenic concentrations were less than 20 *μ*g/L. The aim of the present study was to achieve a proper clinical application of compound-QHP that could improve the efficacy and decreased the adverse events by modifying the dosage of realgar and improving the blood arsenic concentrations.

## 2. Materials and Method

### 2.1. Patients

Inclusion criteria were as follows: (1) patients met the diagnosis and classification of MDS according to the diagnosis of MDS and the WHO MDS criteria in 2007 [7, 8], and the prognosis standard of MDS adopted the revised international prognosis scoring system (IPSS-R) [9]. (2) Patients did not use arsenic-containing drugs for the past 2 months before the enrollment. (3) The age of patients is between 18 and 75 years. (4) Patients must have signed an informed consent form.

Exclusion criteria were as follows: (1) patients with severe diseases in heart, liver, kidney, and peripheral nerves; (2) woman with pregnancy or lactation; (3) patients with severe mental illness; (4) patients in other trials.

A final number of 200 patients who met the diagnosis and classification of MDS were collected; all of them were admitted to the Department of Hematology in Xiyuan Hospital, China Academy of Chinese Medical Sciences, ranging from March 2015 to November 2016. The age of them was between 18 and 72 years and the average age was 56.5 ± 15.5 years. Patients were orally administered with compound-QHP, prepared by Preparation Laboratory of Xiyuan Hospital, at a daily dose of 2.7 g including* realgar* 0.1 g,* indigo naturalis* 0.2 g,* white peony root* 0.96 g,* Atractylodis macrocephala* Koidz 0.48 g,* Radix Ledebouriellae* 0.48 g, and* Pericarpium Citri Reticulatae* 0.48 g.

### 2.2. Experimental Design

The blood arsenic concentration of 200 patients was detected by atomic fluorescence spectrophotometry (HF-AFS) after one month's treatment with compound-QHP. We adopted randomized, single blind, and case control methods to divide them into group A and group B for patients whose arsenic concentrations were less than 20 *μ*g/L after the first month of treatment. Daily dose of 0.1 g realgar was maintained during the course of treatment in group A, while it was increased by 0.1 g each time until the blood arsenic concentrations were more than 20 *μ*g/L in group B. A period of three months was considered as a course of treatment and the patients received 2 courses of treatments. The blood arsenic concentrations were tested during the course of treatment at 1, 3, and 6 months as three time points. The dose of realgar would be reduced by half when the blood arsenic concentrations were more than 140 *μ*g/L, and treatment would be discontinued when the blood arsenic concentrations were more than 940 *μ*g/L, which were determined with reference to the reported peak safe concentrations of patients receiving intravenous injection of arsenous acid. All patients continued to receive the compound-QHP after the end of clinical trials.

Among 200 patients, 148 cases were only treated with compound-QHP; 26 of them had previously been accepted Stanozolol and had stopped at least one month before the treatment with compound-QHP. Another 52 cases were treated with combination of compound-QHP and Stanozolol (2~4 mg/d). Transfusion of blood components was used as the supportive therapy if necessary.

### 2.3. Assessment of Response and Safety Analysis

All patients received assessment of response after the treatment according to IWG 2006 criteria of MDS [10].

The safety assessment of treatment with compound-QHP included two aspects, which were symptoms and organic functions of adverse reactions. Side effect symptoms included nausea, anorexia, abdominal pain, diarrhea, limb numbness, skin keratinization, and swelling.

Adverse reaction grading standards were in accordance with the 2002 Clinical Guideline of New Drugs for Traditional Chinese Medicine: (1) light grade: the patients could tolerate the side effect of treatment and continue the study without other disposition. And it had no significant damage of health. (2) Moderate grade: the patients' health was damaged and they could not tolerate the side effect. (3) Severe grade: the side effect symptom jeopardized the life of the subjects who were disabled or died. They need to stop the treatment and dispose immediately. The organic functions were analyzed by myocardial enzymes, liver functions, and renal functions.

### 2.4. Statistical Analysis

All blood arsenic concentrations were detected in triplicate. Statistical analysis was performed by using GraphPad Prism software version 6.0. Measurement data was compared with test and expressed as mean ± standard deviation. Counting data was compared with chi-square test and expressed as a percentage. *P* < 0.05 was considered to indicate a statistical difference.

## 3. Result

### 3.1. General Characteristics of Patient Population

60 of 200 patients whose blood arsenic was less than 20 *μ*g/L after the first month of treatment were randomly divided into group A (Control group) and group B (Experimental group). Daily dose of realgar was maintained in group A, while that was increased until the blood arsenic concentrations were more than 20 *μ*g/L in group B. Nine patients dropped out by reason of referral (Control group: *N* = 4; Experimental group: *N* = 5), and 3 patients dropped out by reason of poor compliance (Control group: *N* = 2; Experimental group: *N* = 1). Finally, 48 cases were collected in this study (Control group: *N* = 24; Experimental group: *N* = 24).  Table 1 summarized the general characteristics of 48 patients. There were no statistical difference between group A and group B in gender (*P* > 0.05), age (*P* > 0.05), karyotype (*P* > 0.05), classification of WHO (*P* > 0.05), IPSS-R Scores (*P* > 0.05), and the blood arsenic concentrations (*P* > 0.05), respectively.

### 3.2. The Blood Arsenic Concentrations and the Treatment Efficacy

**Figure 1:**
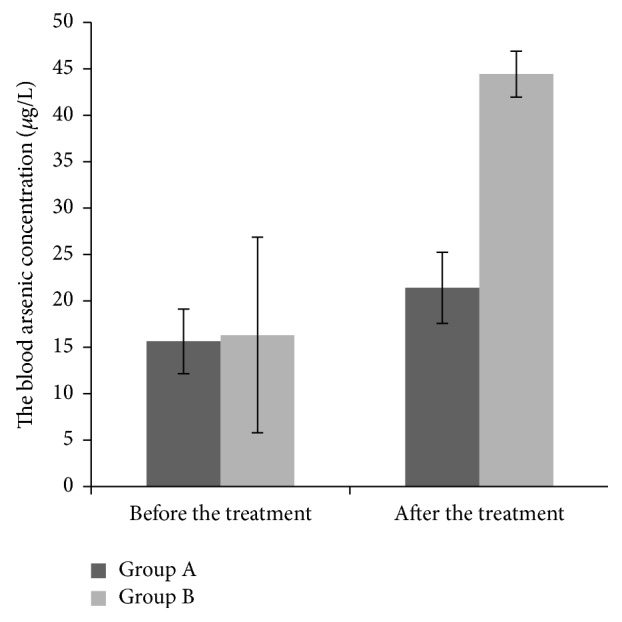
The comparison of blood arsenic concentrations between two groups after the treatment, ^*∗*^*P* < 0.01.

The average blood arsenic concentrations of group A after two courses of the treatment were 21.39 ± 3.49 *μ*g/L, while that in group B increased to 44.43 ± 10.54 *μ*g/L. There was a significant difference between two groups (*P* < 0.01) (Figure 1)

**Figure 2:**
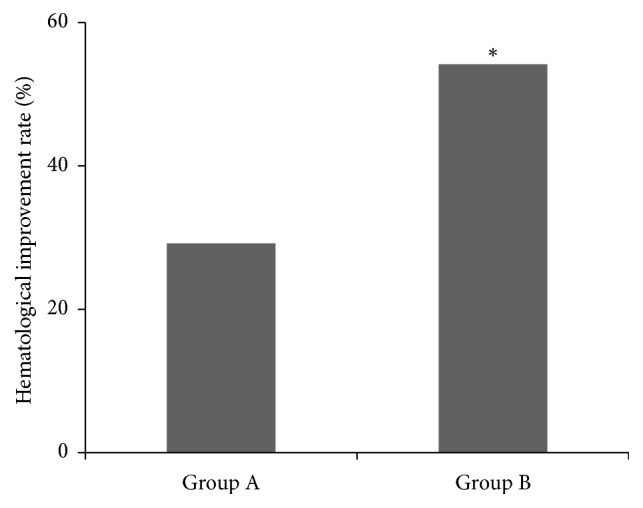
The comparison of hematological improvement rate between group A and group B after the treatment, ^*∗*^*P* < 0.05.

After the treatment, 7 out of 24 patients in group A achieved the hematological improvement; 14 patients were in stable condition while another 3 patients had no response to treatment. Five patients depended on transfusion of red blood cells before the treatment; 1 out of them was in no need for transfusion after the treatment. The rate of hematological improvement was 29.2%. In group B, 13 patients achieved the hematological improvement and 8 patients were in stable condition while another 3 patients had no response to treatment. Seven patients depended on transfusion of red blood cells before the treatment, 5 out of them were independent of transfusion and in 1 patient the interval of transfusion was extended after the treatment. The reduced transfusion rate after the treatment in group B (71.43%) was significantly higher than that in group A (20%) (*P* < 0.05). The rate of hematological improvement in group B was 54.2%, which was significantly higher than that in group A (29.2%) (*P* < 0.05) (Figure 2)

The cell counts including hemoglobin (HGB), absolute neutrophil count (ANC), and platelets (PLT) in peripheral blood of group A had no significant difference after the treatment (*P* > 0.05), while those counts in group B had significantly increased. In group B, the HGB increased from 75.59 ± 24.94 g/L to 86.12 ± 26.35 g/L (*P* < 0.05). The ANC increased from 1.02 ± 0.37  × 10^9^/L to 1.28 ± 0.60  × 10^9^/L (*P* < 0.05), and the PLT increased from 50.11 ± 33.55  × 10^9^/L to 61.89 ± 36.93  × 10^9^/L (*P* < 0.01) (Table 2).

### 3.3. Side Effect

*Clinical Adverse Symptoms*. Two patients who received the treatment in group A had symptoms of diarrhea, and 1 out of 2 patients had a complication of gastric malaise. In group B, 2 patients had symptoms of diarrhea. Overall, the clinical adverse symptoms are light degree, and no patients need to stop the therapy (Table 3).

### 3.4. Organic Function

During the 6-month treatment period, 3 patients in group A were with liver dysfunction before the treatment (2 cases with high level of ALT and 1 case with high level of GGT), while 1 of 3 patients recovered and 2 of 3 patients alleviated after the treatment. In group B, 6 patients were with liver dysfunction (6 cases with high level of ALT) before the treatment, while 2 of 6 patients recovered and 4 of 6 patients alleviated after the treatment. Before treatment, five patients were with increased cardiac enzyme in group A (2 cases with high level of LDH, 4 cases with high level of HBDH) and 1 of 5 patients recovered and 4 of 5 patients alleviated after the treatment. Four patients were with increased cardiac enzyme in group B (4 cases with high level of LDH, 4 cases with high level of HBDH), and 1 of 4 patients recovered and 3 of 4 patients alleviated after the treatment. No patients were with renal dysfunction before the treatment. There were no new cases with abnormal liver functions, cardiac enzymes, and renal functions (Table 4).

## 4. Discussion

There has been a long history of treatment with traditional Chinese arsenic-containing medicine [11], which mainly consists of arsenic trioxide (As_2_O_3_) and realgar (As_2_S_2_). Traditional Chinese arsenic-containing medicine in treating MDS has been a popular area of research recently [2, 3], which is characterized by its better curative efficacy, agreeable price, and less side effect that could be tolerated by patients. Data from the research suggested that the oral dose of realgar (As_2_S_2_/As_4_S_4_) was usually more than 0.9 g/d in the treatment of hematological malignancy, while the dose was normally used as 3 g/d with the blood arsenic concentrations ranging from 20 to 110 *μ*g/L. The mainly adverse reactions of those doses were side digestive effects. The blood arsenic concentration could reach 900 *μ*g/L with the intravenous drip of the As_2_O_3_ at 10 mg per day, while no patients appeared to have severe side effect [12].

QHP, which consisted of natural indigo and realgar, was the effective drug in treating hematological diseases developed by hematology department of Xiyuan Hospital after a long period of clinical practice [2–6, 11]. The properties of realgar were described as warmth, acridity, and toxicity in traditional Chinese medicine. The dose of realgar has been changed several times in Chinese Pharmacopoeia: oral dose of realgar ranged from 0.3 to 1.0 g per day according to version in 1977, from 0.15 to 0.3 g per day according to version in 1990, and from 0.05 to 0.1 g per day according to version in 1995–2015 [13].

Previous studies have suggested that QHP in treating patients with MDS have a great efficacy except some patients with no response [2]. The side effect increased and particularly the digestive reaction of realgar increased randomly for better efficacy. Further study has suggested that the efficacy on patients with QHP was related to the blood arsenic concentrations [4] and enough blood arsenic concentrations were the key to obtain the curative effect in treating MDS. Diarrhea could reduce the absorption of QHP in digestive tract and decrease the efficacy on account of insufficient blood arsenic concentrations.

Compound-QHP have optimized the component of QHP, and* white peony root*,* Atractylodis macrocephala* Koidz,* Radix Ledebouriellae*, and* Pericarpium Citri Reticulatae* were added in the new prescription in addition to* indigo naturalis* and* realgar* to prevent the gastrointestinal adverse reaction. Our previous research suggested that compound-QHP in treating patients with MDS decreased adverse digestive reactions and increased efficacy compared to those who received QHP. The previous in vitro study suggested the active ingredients of realgar (As_2_S_2_) as the monarch drug in compound-QHP that could induce the apoptosis and promote the differentiation in a dose-dependent manner for MDS/AML [14–17].

Our previous studies have showed that the lowest blood arsenic concentration of efficacy is 20 *μ*g/L [4]. In this study, the patients who received compound-QHP with insufficient blood arsenic concentrations (<20 *μ*g/L) were randomly assigned to group A and group B. Daily dose of realgar was maintained in group A, while that was increased until the blood arsenic concentrations were more than 20 *μ*g/L in group B. The concentrations after the treatment in group B were significantly higher than those in group A (44.43 ± 10.54 versus 21.39 ± 3.49 *μ*g/L, *P* < 0.05). The rate of hematology improvement was significantly higher in group B than that in group A (54.2% versus 29.2%, *P* < 0.05). The study suggested that increasing the blood arsenic concentrations is important to develop the efficacy with compound-QHP. The greater dose (0.2 g/d) of realgar could increase the concentrations in treating MDS without more side effects. The increased concentration is significantly lower than that in other methods of usage with arsenical in literature [12]. The patients of the two groups were both followed for 3 months after the six-month treatment.

There were few adverse digestive reactions with light grade in patients with compound-QHP, and those were gradually decreased with the treatment in some patients. The result suggested that patients with the treatment of compound-QHP could tolerate the side effect and there is no need to stop the therapy. Some patients with liver dysfunction and abnormal cardiac enzyme are related to testosterone and long-term of anemia, respectively, and some of them could recover after the treatment. There is no patient with renal dysfunction before the treatment, and there are no new cases with abnormal liver function, cardiac enzyme, and renal function during the treatment. Overall, compound-QHP is safe for organic function, which is consistent with previous study.

## 5. Conclusions

Monitoring the blood arsenic concentration and adjusting the daily dose of realgar in order to increase the effective blood arsenic concentration and improving efficacy without increasing the clinical toxicity are the safe and effective clinical methods in the treatment of MDS.

## Figures and Tables

**Table 1:** General characteristics of 48 patients treated with compound-QHP.

Group	Group A *N* = 24	Group B *N* = 24
Gender [*N*/(%)]		
Male	7 (29.2)	10 (41.7)
Female	17 (70.8)	14 (58.3)

Average age (years)	51.9 ± 16.7	52.4 ± 14.5

Karyotype risks		
Very good/good	12 (50)	10 (41.67)
Intermediate	7 (29.17)	8 (33.33)
Poor/very poor	5 (20.84)	6 (25.0)

Diagnosis according to WHO [*N*/(%)]		
RA/RCMD/RARS	20 (83.3)	19 (79.2)
RAEB1	3 (12.5)	3 (12.5)
RAEB2	1 (4.2)	2 (8.4)

IPSS-R score [*N*/(%)]		
Very low/low	5 (23.8)	6 (25.0)
Intermediate	17 (70.8)	16 (66.7)
high/very high	2 (8.3)	2 (8.3)

The blood arsenic concentrations after one month of treatment (*µ*g/L)	15.63 ± 3.83	16.31 ± 2.47

**Table 2:** The comparison of the cell counts in peripheral blood between two groups after the treatment.

Group	Time	ANC < 1.5 (×10^9^/L)	HGB < 120 (g/L)	PLT < 125 (×10^9^/L)
A	Before the treatment	0.90 ± 0.41	74.00 ± 16.16	56.95 ± 34.96
	After the treatment	1.15 ± 0.60	78.17 ± 22.02	57.00 ± 34.23
B	Before the treatment	1.02 ± 0.37	75.59 ± 24.94	50.11 ± 33.55
	After the treatment	1.28 ± 0.60^*∗*^	86.12 ± 26.35^*∗*^	61.89 ± 36.93^*∗*^

In group B, the levels of HBG, ANC, and PLT after treatment were significantly higher than that before treatment, respectively. _ _^*∗*^*P* < 0.05.

**Table 3:** Clinical adverse symptoms of group A and group B.

Symptoms	Number of patients (%)	
	Group A	Group B
Gastric malaise	1 (4.17)	0
Anorexia and nausea	0	0
Diarrhea	2 (8.33)	2 (8.33)
Numbness of limbs	0	0
Skin keratosis	0	0
Edema	0	0

**Table 4:** Abnormal organic function between group A and group B (number of patients).

Organic function	Indicator	Group A		Group B	
		BTT	ATT	BTT	ATT
Liver functions	ALT	2	2	6	4
	AST	0	0	0	0
	GGT	1	0	0	0

Cardiac enzymes	LDH	2	2	4	3
	HBDH	4	3	4	3
	CK-MB	0	0	0	0

Renal functions	CRE	0	0	0	0
	BUN	0	0	0	0
